# The Influence of Accommodation on Retinal Peripheral Refraction Changes in Different Measurement Areas

**DOI:** 10.1155/2023/5553468

**Published:** 2023-05-22

**Authors:** Weicong Lu, Zisu Peng, Wenzhi Ding, Rongyuan Ji, Yuyin Tian, Chenpei Zhao, Lin Leng

**Affiliations:** ^1^Qingdao Eye Hospital of Shandong First Medical University, Qingdao, China; ^2^State Key Laboratory Cultivation Base, Shandong Provincial Key Laboratory of Ophthalmology, Eye Institute of Shandong First Medical University, Qingdao, China; ^3^Department of Ophthalmology & Clinical Center of Optometry, Peking University People's Hospital, Beijing 100044, China; ^4^College of Optometry, Peking University Health Science Center, Beijing, China; ^5^Eye Disease and Optometry Institute, Peking University People's Hospital, Beijing, China

## Abstract

**Background:**

The change in refraction caused by accommodation inevitably affects the peripheral defocus state and thus may influence the effect of retinal peripheral myopic defocus measures in myopia control. This study investigated accommodation changes in different peripheral retinas under cycloplegia to help improve myopia control.

**Methods:**

Fifty-six eyes of fifty-six myopic subjects were recruited for this prospective study. The center and peripheral retina refractions were measured using multispectral refractive topography. The subjects were divided into low-to-moderate myopia group (range: −1.25 D to −6.00 D) and high myopia group (range: −6.25 D to −9.75 D) according to spherical equivalent (SE). The compound tropicamide (0.5% tropicamide and 0.5% phenylephrine) was used to relax the accommodation. The difference between cycloplegia and non-cycloplegia peripheral retinal refraction was analyzed using the *t*-test. The correlation between eccentricity and changes in peripheral refraction was analyzed using Pearson's correlation analysis.

**Results:**

The manifest refraction of the retina significantly decreased with an increase in eccentricity after cycloplegia. The annular refraction difference value at 50°–53° (ARDV 50–53) showed the largest refraction decrease of 1.31 D compared with the central retinal refraction decrease of 0.84 D. The inferior quadrantal refraction difference value had the least change compared to the other quadrants. The relative peripheral refraction (RPR) changes in refraction difference value (RDV) at 15° (RDV-15), RDV-30, and RDV-45 were less than 0.15 D. When the range of annulus narrowed to 5°, the narrower annulus showed faster change with eccentricity increase in ARDV 30–35, ARDV 35–40, ARDV 40–45, ARDV 45–50, and ARDV 50–53. The RPR was highly correlated with eccentricity (*R* = 0.938 and *P* < 0.001). The high myopia group had a greater hyperopic shift in the periphery than the low-to-moderate group after cycloplegia.

**Conclusions:**

Peripheral refraction showed a significant hyperopic shift after cycloplegia with an increase in eccentricity. The RPR became more hyperopic than the central refraction. The high myopia group showed more hyperopic shifts in the peripheral region. Accommodation should be taken into consideration in peripheral defocus treatment.

## 1. Introduction

With the prevalence of myopia worldwide, myopia is becoming a worldwide health problem that influences 80% of the population [1], especially in Asia, where the incidence of myopia in adolescents reaches nearly 90% [2, 3]. Previous reports provided a reliable theory for the mechanism of controlling myopia, indicating that the peripheral defocus state may be the key to myopia progression [4]. Peripheral hyperopic defocus can prompt the progress of myopia, while peripheral myopic defocus can delay myopia progress. This makes the change of peripheral myopic defocus an excellent method for myopia control [5]. The current clinically available treatment for myopia control is to manually create a peripheral myopic defocus to prevent axial length (AL) elongation.

The most widely used methods for myopia control are orthokeratology, multifocal soft contact lenses (MFSCL), and defocus incorporated multiple segments (DIMS) spectacles. The effect of orthokeratology and defocus lens has been proven to slow the AL elongation by 30%–62% [6, 7]. However, Sankaridurg et al. discovered that there was no difference between DIMS spectacles, peripheral myopic contact lens, and single vision (SV) lens in myopia control [8]. Lam et al. conducted a double-masked randomized study about the difference between DIMS group and SV group in myopic control of Chinese children [9]. They highlighted that the children who wore DIMS spectacles showed 52% slower refractive progression and 62% slower AL elongation. Other studies that analyzed the effectiveness of designed peripheral defocus lenses also showed contradictory results in refractive progression and AL elongation [10–12]. The contradictory outcomes regarding designed peripheral defocus lenses have not been resolved. These results may be due to the differences in the designs of peripheral defocus lenses that lead to a diversity of peripheral defocus in the retina, as standard peripheral myopic defocus remains unknown; this could explain the differences in myopia control effects. In daily life, as the eyes focus on objects at different distances, the lens adjusts to see the target, especially for school-age children. Close reading is the most common way to use the eyes. The change in refraction caused by accommodation inevitably affects the peripheral defocus state. However, the changes in refraction in the center and periphery of the retina during accommodation have not been fully elucidated. Several studies have analyzed the influence of accommodation on the change of peripheral refraction; however, the device they used for peripheral refraction measurement could only obtain the horizontal off-axis refraction with six located points. The separated point could not represent the zonal peripheral refraction state, and the area of peripheral refraction may play a better role than point in myopia progression [13].

To our knowledge, the most widely used device for peripheral defocus measurement in clinics is the WAM-5500 (Grand Seiko Co., Hiroshima, Japan). It can provide repeatable results in central and peripheral defocus [14]. However, the limitations of the measurement region and site requirements make it not an ideal device to measure peripheral defocus at any location. We have reported that the novel multispectral refractive topography (MRT, Thondar, Inc., China) can measure peripheral refractive error in different zones with good repeatability [15]. The large measurement area of the peripheral retina makes it a better choice for peripheral refraction analysis with different optical treatments. However, the peripheral defocus with different accommodation states of the eye cannot be measured with current devices, as exact accommodation stimulation cannot be carried out. Instead, we attempted to analyze the differences in refraction between the normal and relaxed states after cycloplegia to indirectly investigate the changes in peripheral refraction caused by accommodation. The findings might help improve the effect of peripheral defocus technology in myopia control by informing the design of individual peripheral refraction according to different vision demands instead of a unified positive power.

## 2. Materials and Methods

This prospective research included 56 myopic subjects (28 males and 28 females) who reported to the Qingdao Eye Hospital of Shandong First Medical University for regular examination between July and August 2021. Only the right eye was selected for this study, owing to the consistency of right and left eyes. The research protocol adhered to the tenets of the Declaration of Helsinki and was approved by the Ethics Committee of the Qingdao Eye Hospital of Shandong First Medical University (QYLS [2021] no. 21). Informed consent was provided after the purpose of the study and the potential risks were presented to subjects. The subjects were divided into two groups according to the spherical equivalent (SE) measured by subjective refraction (NIDEK AOS1500 + SSC3): low-to-moderate group (−6.00 D ≤ SE ≤ −0.50 D) and high myopia group (SE < −6.00 D). The inclusion criteria for subject screening were as follows: older than 18 years, astigmatism and anisometropia up to 1.50 D, without any active ocular pathology or any previous surgery, without systemic abnormalities, and without a history of any ocular trauma or undergoing any myopia control techniques.

Central and peripheral refractions were measured using MRT. The refraction of the central and peripheral retinas was first measured three times before cycloplegia. Then, compound tropicamide (0.5% tropicamide and 0.5% phenylephrine, SINQI Pharmaceutical Co., Ltd., Shenyang, China) was used three times with an interval of 5 min to relax the accommodation. After 30 min, when the pupil diameter reached 8 mm, the subjects were asked again to sit behind the MRT, and the central and peripheral refractions were recorded three times. This procedure was carried out between 10:00 AM and 12:00 PM in a dim room by one experienced operator to avoid the potential influence of diurnal variation [16].

**Figure 1:**
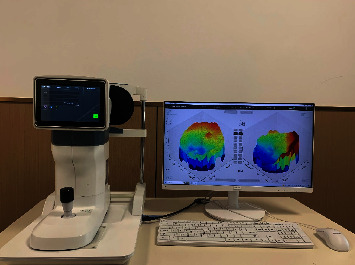
A physical image of the multispectral refractive topography (MRT, Thondar, Inc., China).

MRT is a novel multispectral imaging technology based on a simplified reduced optical model. It can compensate for the blur retinal image to clear the image using a refractive compensation system (Figure 1). The compensated refraction power represents the refractive state of the retina. The compensation refraction is regulated according to the change in the focal length of the fundus camera, which can adjust the retina image formed on the sensor plane. After obtaining the sharping profile of the image, the internal image analysis system was used to decouple and generate the refractive value of each image. Through this approach, 128 × 128 points on 53° of the retina can be detected. The acquired data points are further processed by the compensation software to form a color-coded image that shows the refraction state in different areas of the retina.

The analyzed parameters were divided into three different types: (1) the refraction difference value (RDV) of circle areas centered on macular with an increment of 15°, RDV-15, RDV-30, RDV-45, and total refraction difference value (TRDV), which indicates the average peripheral retinal refraction from the center to 15°, 30°, 45°, and total peripheral retina refraction (including the fovea); (2) the annular refraction difference value (ARDV) with intervals of 15° or 5°, ARDV 15–30, ARDV 30–45, ARDV 0–5, ARDV 5–10, ARDV 10–15, ARDV 15–20, ARDV 20–25, ARDV 25–30, ARDV 30–35, ARDV 35–40, ARDV 40–45, and ARDV 45–50, which indicates the average refraction of the concentric areas with different angles (the maximum measurement range of MRT is 53°, and the ARDV45–53 and ARDV 50–53 represent the most peripheral annular data); and (3) the quadrant of the retina which was defined as inferior, superior, nasal, and temporal (QRDV-I, QRDV-S, QRDV-N, and QRDV-T). To ensure the reliability of the measurement, all the measures were conducted three times, avoiding the influence of tear quality, eye blinking, and iris reflection. Only measurements showing quality scores above 80% were recorded.

### 2.1. Statistical Analysis

The statistical software used for the data analysis was SPSS software (version 24.0; IBM Corporation, Armonk, NY). Before analysis, the normality of the data was checked by the Kolmogorov–Smirnov test, and all the data were normally distributed (*P* > 0.05). The profile of the data was expressed as mean ± standard deviation (SD). The difference between cycloplegic and non-cycloplegic peripheral retinal refraction was analyzed by the paired *t*-test, as well as the change in relative peripheral refraction (RPR). The difference between the low-to-moderate myopia group and the high myopia group was analyzed using the independent samples *t*-test. The equality of variance was checked by Levene's test. The correlation between eccentricity and changes in peripheral refraction was analyzed using Pearson correlation analysis. *P* < 0.05 was considered statistically significant.

## 3. Results

The mean age of fifty-six subjects was 27.95 ± 6.77 years (range: 19–43 years). The mean SE before cycloplegia was −5.53 ± 1.74 D and −5.21 ± 1.83 D after cycloplegia.

Table 1 shows the refraction state of the retina before and after cycloplegia. After treatment with compound tropicamide, the manifest refraction of the retina significantly decreased with an increase in eccentricity. The ARDV 50–53 showed the largest refraction decrease and was nearly 1.5 times than the change of central refraction. Peripheral refraction within 30° showed a similar change compared with central refraction. Further, the change in manifest refraction in QRDV was larger than central refraction. The ARDV 0–5, ARDV 5–10, and ARDV 10–15 are similar to central refraction. The correlation between eccentricity and the relative change ratio was further analyzed using Pearson's correlation analysis. This showed that they were highly correlated (*R* = 0.938 and *P* < 0.001). With respect to changes in quadrant refraction, all four quadrants showed significant changes after cycloplegia compared to central refraction (*P* < 0.05).

**Figure 2:**
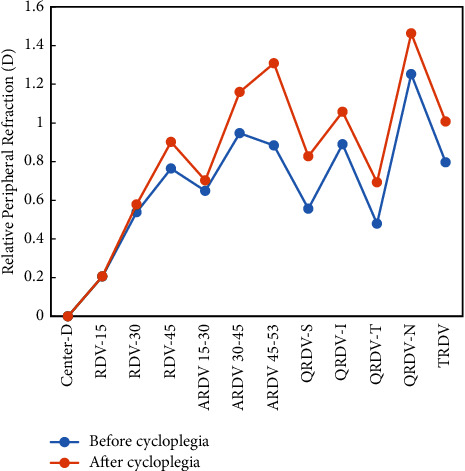
The change in relative peripheral refraction for different measurement areas on the retina. TRDV, total peripheral retina refraction; RDV, refraction difference value; ARDV, annular refraction difference value; QRDV, quadrant refraction difference value; S, superior; I, inferior; N, nasal; T, temporal.

**Figure 3:**
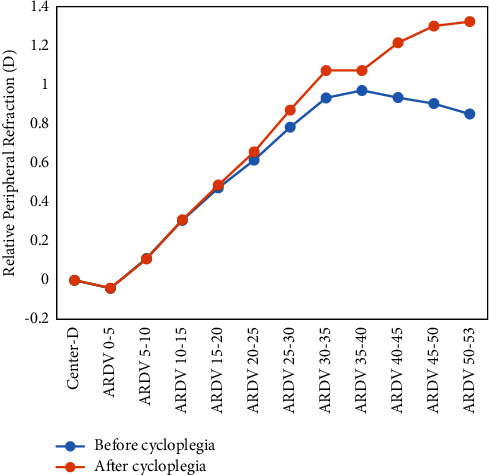
The change in relative peripheral refraction. RDV, refraction difference value; ARDV, annular refraction difference value.

Table 2 shows the RPR variation after cycloplegia treatment. As shown in Table 2, the RPR change in RDV-15, RDV-30, and RDV-45 was less than 0.15 D. The ARDV 15–30, ARDV 30–45, and ARDV 45–53 showed that the RPR change increases with eccentricity, although the ARDV 15–30 was slightly lower (0.05 D). When the interval of measurement area was reduced to 5°, the narrower annulus showed faster change with eccentricity increase compared with 15° annulus in ARDV 30–35, ARDV 35–40, ARDV 40–45, ARDV 45–50, and ARDV 50–53. There was a significant positive correlation between RPR and eccentricity (*R* = 0.904 and *P* < 0.001). Figures 2 and 3 show the RPR value under different regions before and after cycloplegia.  Figure 2 shows that the RPR after cycloplegia was slightly larger than before cycloplegia; the change was mild, except for the ARDV 45–53, with a change of 0.42 D.  Figure 3 shows that cycloplegia induced a significant hyperopic shift, with ARDV 50–53 showing the largest RPR hyperopic shift of 0.47 D, whereas the change of RPR in ARDV 0–5, ARDV 5–10, ARDV 10–15, and ARDV 15–20 showed no difference. However, the difference in RPR after cycloplegia showed a hyperopic shift and increased with eccentricity. We further analyzed the correlation between change in RPR and SE, which showed that the RPR significantly changes in TRDV, RDV-45, ARDV 30–45, QRDV-S, QRDV-T, ARDV 30–35, ARDV 35–40, ARDV 40–45, ARDV 45–50, and ARDV 45–53 and was negatively correlated with SE (Table 3), and this indicates that the RPR change above 30° was correlated with SE and so were the superior and temporal quadrants (Table 3).

We also analyzed the RPR change in the low-to-moderate myopia group and the high myopia group.  Table 4 demonstrates the difference of the different groups. There was a statistically significant difference in RPR at TRDV, RDV-45, ARDV 30–45, ARDV 45–53, QRDV-S, QRDV-T, ARDV 30–35, ARDV 35–40, ARDV 40–45, ARDV 45–50, and ARDV 50–53 between low-to-moderate myopic group and high myopic group after cycloplegia. This suggests that the high myopia group had a greater hyperopic shift in peripheral refraction than the low-to-moderate myopia group after cycloplegia. The variation was more significant in the peripheral region as eccentricity increased, and QRDV-S and QRDV-T showed the same change. However, the RPR before cycloplegia showed that these two groups had no statistical difference (*P* > 0.05).

## 4. Discussion

Given the increasing prevalence of myopia, myopia is becoming a worldwide health and economic problem [1]. Optical means are the most widely used approaches for myopia control based on the peripheral refraction that transforms the peripheral hyperopic state into the peripheral myopic state [4, 13]. However, the amount of peripheral myopic defocus that can effectively control the progression of myopia is still unknown. Further, the different accommodation states in daily work can also influence peripheral refraction. Currently, peripheral refraction can be measured by several devices such as WAM-5500 and Shin-Nippon NVision K5001 from specific points and the peripheral refraction of larger area is unavailable. In our study, we used the cycloplegia to simulate changes in refractive status under accommodation [17], even though the refraction change based on cycloplegia may not represent the actual accommodation state in daily life. The accommodation response trend could also be indirectly observed to some extent with this method.

The influence of cycloplegia on central and peripheral refraction showed that the different regions of the retina had different reactions to cycloplegia. The change in retinal refraction increased with eccentricity, and it was the largest at the area of ARDV 50–53. After cycloplegia, the peripheral defocus varied more than the center. Previous studies reported that hyperopes and emmetropes have a negative relative peripheral refraction, while myopes tend to be more positive in peripheral refraction [18]. Whatham et al. studied the influence of accommodation on peripheral refraction in myopes using an autorefractor with a custom near-fixation target [19]. They found that the SE of the peripheral retina was more hyperopic relative to central refraction at all eccentricities, except the temporal retina at 20° and 30° at distance. Although the 40 cm and 30 cm target induced more positive refraction in nasal and more negative refraction in temporal, the accommodation level was consistent with an increase in eccentricity. Lundström et al. used a Hartmann–Shack wavefront sensor to assess the change in peripheral refraction under accommodation [20]. They discovered that the peripheral refraction in myopia only showed an inconsistent change between far and near vision, while the emmetropia had a myopia shift in peripheral refraction. However, Davies and Mallen reported that the temporal retina exhibited a significant hyperopic shift with increasing eccentricity, but the increasing accommodation demands did not influence the peripheral refraction [21]. Our findings indicate that the peripheral refraction was more positive than central refraction, and the tendency of the hyperopic defocus of the peripheral retina was more significant when the measurement region became smaller.

Compared to peripheral refraction, the RPR may be a better parameter to evaluate the peripheral retinal refraction state and myopia progression control. Our results showed that the RPR change under cycloplegia was correlated with eccentricity. The change in RDV-15, RDV-30, and RDV-45 was less than ARDV 15–30, ARDV 30–45, and ARDV 45–53; differences in these parameters may be due to their various definitions as well as variations in measurement regions. RDV-15, RDV-30, and RDV-45 measure the circle area, including the foveal refraction, while ARDV 15–30, ARDV 30–45, and ARDV 45–53 only measure the annular area of the retina without the foveal refraction. As the foveal refraction is calculated to normalize the RPR, the average RPR change, including the foveal refraction, will significantly decrease the change amplitude of the power. Queiros et al. measured the center, 20°, and 40° of retinal refraction with accommodation target set at 2.00, 0.50, 0.33, and 0.20 m [13]. They found that the spherical component of the RPR was significantly different at 40° temporal compared with central refraction at a target distance of 2.00 m. The change of RPR was larger at 40° temporal than at central, with an accommodation from 2.00 m to 0.20 m. Accommodation stimulation may have more influence on RPR changes with the increase of eccentricity. Similar results were also described by Whatham et al. who set three levels of accommodation [19]. The authors proved that the RPR change was a more myopic shift under accommodation from 2.00 m to 0.3 m, and the change amplitude increased with eccentricity. However, the accommodation stimulation from 0.40 m to 0.30 m only induced less change of RPR at 40° peripheral. Although we did not draw the same conclusion, direct accommodation was unavailable for our study. The difference may be that the compound tropicamide used for cycloplegia could induce more accommodation changes than the near target stimulation. The cycloplegia could have a more relaxing function in peripheral than central, which made the RPR more hyperopic in peripheral. This suggests that the RPR was more liable to relaxation as eccentricity increased, which proved that the RPR may have more influence on peripheral defocus treatment for myopia control. A variety of spectacle lenses with peripheral defocusing capability known as defocus incorporated multiple segments (DIMS) technology have been marketed since 2021 [9]. The design of peripheral defocus treatment must consider the influence of accommodation in the RPR and add additional defocus power.

The differences in refractive errors in various regions of the retina among different myopia groups have also been studied by several researchers. Sng and colleagues studied peripheral refraction error in Singapore Chinese children with different refractive error [22]. They found that for high and moderate myopia, RPR in temporal 30°, temporal 15°, nasal 30°, and nasal 15° was 1.23 D, 0.29 D, 0.25 D, and 1.93 D, respectively. For low myopia, the values were 0.009 D, −0.18 D, −0.16 D, and 0.50 D, respectively, and for emmetropia, they were −0.43 D, −0.44 D, −0.56 D, and −0.19 D, respectively. There was a significant difference between the two myopia groups. Nevertheless, we studied peripheral refraction before cycloplegia and found that the difference was insignificant between low-to-moderate myopia group and high myopia group. This may be because the subjects included in our study were adults aged 19 to 44 years, whereas the other studies recruited children. The change in retinal shapes accompanying the progression of myopia results in variations in peripheral defocus, whereas the profile of the retina in adults is stable, as is the RPR. We further analyzed the changes in RPR after cycloplegia in different groups. The results showed that cycloplegia induced the RPR to be more hyperopic in the high myopia group than in the low-to-moderate myopia group. A significant difference was observed in ARDV 45–53, ARDV 45–50, ARDV 50–53, QRDV-S, and QRDV-T. Lundström et al. measured peripheral refraction under accommodation demands of 0.50 D and 4.00 D [20]. They found that, when compared with emmetropes, myopes became relatively more hyperopic in peripheral refraction, although myopes did not show a consistent change in peripheral refraction. Mathur et al. showed that the RPR was stable under accommodation stimulation of 0.30 D and 4.00 D [23]. Similar findings have been reported by Queiros who demonstrated that the myopes showed a more significant hyperopic shift than emmetropes; when the accommodation demands changed from 0.20 m to 2.00 m, the RPR change was statistically significant in temporal 40° [13]. The RPR was more hyperopic in the low accommodation stimulation. Although we used a different method, our study drew a similar conclusion: subjects with high myopic power had larger hyperopic change after cycloplegia than the low-to-moderate myopia group.

The difference in RPR change with eccentricity increase made the peripheral refraction beyond 40° and temporal and superior quadrants better parameters for peripheral refraction and myopia control research under accommodation. The commonly used myopic adding lenses were +2.00 to +3.50 D for multifocal soft contact lenses and DIMS spectacles [9, 24]; however, the largest difference value of ARDV 50–53 was up to 0.47 D. The ratio of the change in peripheral refraction and adding power was about 20%; daily accommodation under different working distances should be taken into consideration to obtain a good myopia control effect.

However, our study has several limitations. First, the subjects included in this study were all adults. Children with more accommodation power are considered a better choice for peripheral refraction and myopia control. Second, in the subgroup of the study based on the refractive error of −6.00 D, the low myopia and moderate myopia subjects were combined into one group. A limitation of the device used in the study is the current unavailability of the measurement of peripheral refraction under the accommodation state. Therefore, larger samples including different refraction errors should be adopted in future studies, and the recommendations for manufacturers regarding the accommodation target are in progress.

## 5. Conclusion

Peripheral refraction exhibited a significant hyperopic shift after cycloplegia in this study, and the change amplitude in peripheral refraction was larger than that of central refraction with an increase of eccentricity. The RPR became more hyperopic than central, especially in ARDV 40–45, ARDV 45–50, and ARDV 50–53. The quadrant region also showed that the QRDV-S was most influenced by cycloplegia. The high myopia group showed more hyperopic shift in peripheral refraction than the low-to-moderate myopia group. The accommodation of subjects should be taken into consideration when adopting peripheral defocus treatment for myopia control.

## Figures and Tables

**Table 1:** The difference in retinal refraction before and after cycloplegia.

	Before cycloplegia		After cycloplegia		
	Mean	Standard deviation	Mean	Standard deviation	Difference
Center-D	−5.53	1.75	−4.69	1.86	0.84
TRDV	−4.74	1.73	−3.69	1.81	1.05
RDV-15	−5.33	1.74	−4.49	1.85	0.84
RDV-30	−5.00	1.72	−4.11	1.82	0.89
RDV-45	−4.77	1.73	−3.79	1.81	0.98
ARDV 15–30	−4.89	1.72	−3.99	1.81	0.9
ARDV 30–45	−4.59	1.75	−3.53	1.81	1.06
ARDV 45–53	−4.65	1.77	−3.38	1.85	1.27
QRDV-S	−4.98	1.73	−3.87	1.83	1.11
QRDV-I	−4.64	1.77	−3.64	1.85	1.00
QRDV-T	−5.06	1.90	−4.00	1.88	1.06
QRDV-N	−4.28	1.74	−3.23	1.92	1.05
ARDV 0–5	−5.57	1.75	−4.73	1.87	0.84
ARDV 5–10	−5.42	1.74	−4.58	1.86	0.84
ARDV 10–15	−5.23	1.74	−4.38	1.85	0.85
ARDV 15–20	−5.06	1.73	−4.21	1.83	0.85
ARDV 20–25	−4.92	1.71	−4.04	1.81	0.88
ARDV 25–30	−4.75	1.72	−3.82	1.81	0.93
ARDV 30–35	−4.60	1.74	−3.62	1.81	0.98
ARDV 35–40	−4.56	1.75	−3.52	1.81	1.04
ARDV 40–45	−4.60	1.75	−3.48	1.82	1.12
ARDV 45–50	−4.63	1.77	−3.39	1.84	1.24
ARDV 50–53	−4.68	1.78	−3.37	1.87	1.31

Center-D, central refraction; TRDV, total peripheral retina refraction; RDV, refraction difference value; ARDV, annular refraction difference value; QRDV, quadrant refraction difference value; S, superior; I, inferior; N, nasal; T, temporal.

**Table 2:** The change in relative peripheral refraction after cycloplegia treatment.

	Mean	Standard deviation	95% CI		*P* value
			Lower	Upper	
TRDV	0.21	0.30	0.130	0.292	**≤0.001**
RDV-15	0.00	0.04	−0.010	0.011	0.946
RDV-30	0.04	0.13	0.007	0.074	**0.020**
RDV-45	0.14	0.24	0.072	0.202	**≤0.001**
ARDV 15–30	0.05	0.16	0.013	0.097	**0.011**
ARDV 30–45	0.21	0.35	0.120	0.307	**≤0.001**
ARDV 45–53	0.42	0.52	0.286	0.563	**≤0.001**
QRDV-S	0.27	0.45	0.150	0.391	**≤0.001**
QRDV-I	0.17	0.46	0.045	0.291	**0.008**
QRDV-T	0.21	0.48	0.087	0.342	**0.001**
QRDV-N	0.21	0.39	0.108	0.314	**≤0.001**
ARDV 0–5	0.00	0.02	−0.004	0.005	0.669
ARDV 5–10	0.00	0.03	−0.011	0.006	0.563
ARDV 10–15	0.00	0.05	−0.012	0.017	0.729
ARDV 15–20	0.01	0.09	−0.010	0.039	0.233
ARDV 20–25	0.04	0.15	0.002	0.082	**0.038**
ARDV 25–30	0.09	0.21	0.031	0.145	**0.003**
ARDV 30–35	0.14	0.28	0.066	0.215	**≤0.001**
ARDV 35–40	0.10	0.30	0.021	0.184	**0.015**
ARDV 40–45	0.28	0.42	0.169	0.391	**≤0.001**
ARDV 45–50	0.40	0.50	0.263	0.531	**≤0.001**
ARDV 50–53	0.47	0.55	0.325	0.621	**≤0.001**

TRDV, total peripheral retina refraction; RDV, refraction difference value; ARDV, annular refraction difference value; QRDV, quadrant refraction difference value; S, superior; I, inferior; N, nasal; T, temporal. The bold values indicate statistical difference (*P* < 0.05).

**Table 3:** The correlation between spherical equivalent and change in RPR.

Parameters	*r*	*P*
TRDV	−0.285	**0.033**
RDV-15	−0.151	0.266
RDV-30	−0.231	0.086
RDV-45	−0.282	**0.035**
ARDV 15–30	−0.236	0.08
ARDV 30–45	−0.285	**0.033**
ARDV 45–53	−0.269	**0.045**
QRDV-S	−0.271	**0.043**
QRDV-I	−0.079	0.56
QRDV-T	−0.391	**0.003**
QRDV-N	−0.017	0.902
ARDV 0–5	0.086	0.529
ARDV 5–10	−0.067	0.623
ARDV 10–15	−0.180	0.184
ARDV 15–20	−0.195	0.149
ARDV 20–25	−0.220	0.103
ARDV 25–30	−0.247	0.066
ARDV 30–35	−0.281	**0.036**
ARDV 35–40	−0.274	**0.041**
ARDV 40–45	−0.280	**0.036**
ARDV 45–50	−0.274	**0.041**
ARDV 50–53	−0.253	0.06

TRDV, total peripheral retina refraction; RDV, refraction difference value; ARDV, annular refraction difference value; QRDV, quadrant refraction difference value; S, superior; I, inferior; N, nasal; T, temporal. The bold values indicate statistical difference (*P* < 0.05).

**Table 4:** The change in RPR between the low-to-moderate myopia group and high myopia group.

	Mean difference	Standard deviation	95% CI		*P* value
			Lower	Upper	
TRDV	0.19	0.08	0.037	0.352	**0.016**
RDV-15	0.01	0.01	−0.010	0.032	0.293
RDV-30	0.06	0.03	−0.006	0.126	0.078
RDV-45	0.15	0.06	0.025	0.278	**0.019**
ARDV 15–30	0.08	0.04	−0.007	0.159	0.073
ARDV 30–45	0.22	0.09	0.043	0.404	**0.016**
ARDV 45–53	0.32	0.13	0.046	0.584	**0.022**
QRDV-S	0.30	0.12	0.065	0.530	**0.013**
QRDV-I	0.06	0.12	−0.188	0.312	0.621
QRDV-T	0.32	0.12	0.071	0.563	**0.012**
QRDV-N	0.11	0.10	−0.098	0.318	0.296
ARDV 0–5	0.00	0.00	−0.010	0.007	0.698
ARDV 5–10	0.01	0.01	−0.011	0.023	0.510
ARDV 10–15	0.02	0.01	−0.011	0.046	0.236
ARDV 15–20	0.03	0.02	−0.013	0.083	0.153
ARDV 20–25	0.07	0.04	−0.012	0.145	0.098
ARDV 25–30	0.11	0.06	−0.002	0.222	0.055
ARDV 30–35	0.17	0.07	0.026	0.314	**0.022**
ARDV 35–40	0.19	0.07	0.042	0.339	**0.013**
ARDV 40–45	0.27	0.11	0.053	0.482	**0.015**
ARDV 45–50	0.32	0.13	0.057	0.575	**0.017**
ARDV 50–53	0.31	0.14	0.020	0.599	**0.036**

TRDV, total peripheral retina refraction; RDV, refraction difference value; ARDV, annular refraction difference value; QRDV, quadrant refraction difference value; S, superior; I, inferior; N, nasal; T, temporal. The bold values indicate statistical difference (*P* < 0.05).

## Data Availability

The data used to support the findings of this study are included within the article.
